# Interaction of avian influenza virus NS1 protein and nucleolar and coiled-body phosphoprotein 1

**DOI:** 10.1007/s11262-012-0849-z

**Published:** 2012-11-28

**Authors:** Chunyu Zhu, Fangliang Zheng, Tingting Sun, Yanting Duan, Jingzhen Cao, Huawei Feng, Lingling Shang, Ying Zhu, Hongsheng Liu

**Affiliations:** 1 Key Laboratory of Animal Resource and Epidemic Disease Prevention, Life Science School of Liaoning University, Shenyang, 110036 China; 2 Research Center for Computer Simulating and Information Processing of Bio-Macromolecules of Liaoning, Shenyang, 110036 China; 3 State Key Laboratory of Virology, Wuhan University, Wuhan, 430072 China; 4 Bio Integration Technology Co., Ltd., Dalian, 116025 China

**Keywords:** Avian influenza virus, NS1, NOLC1, Protein–protein interaction

## Abstract

Nonstructural protein 1 (NS1) is a non-structural protein of avian influenza virus. It can interact with a variety of proteins of the host cells, enhancing the expression of viral proteins and changing the growth and metabolism of the host cells, thereby enhancing the virus’ pathogenicity and virulence. To investigate whether there are more host proteins that can interact with NS1 during viral infection, T7-phage display system was used to screen human lung cell cDNA library for proteins that could interact with NS1. One positive and specific clone was obtained and identified as nucleolar and coiled-body phosphoprotein 1(NOLC1). The interaction between these two proteins was further demonstrated by His-pull-down and co*-*immunoprecipitation experiments. Co-expression of both proteins in HeLa cell showed that NS1 and NOLC1 were co-localized in the cell’s nucleus. Gene truncation experiments revealed that the effector domain of NS1 was sufficient to interact with NOLC1. The results demonstrated a positive interaction between a viral NS1 and NOLC1 of the host cells, and provided a new target for drug screening.

## Introduction

The recent emergence of highly pathogenic avian influenza viruses (H5N1) have raised significant global health concerns because of the epizootic and panzootic nature of these viruses, as well as their association with lethal human infections [1, 2]. Influenza virus contains eight RNA segments, the eighth of which encodes two proteins: NS1 (nonstructural protein 1) and NS2/NEP (nonstructural protein 1/nuclear export protein). NS1 is a multifunctional protein of 202–237 amino acids with a molecular weight of about 28 kDa that is translated from unspliced mRNA [3, 4]. Two functional domains have been identified in the NS1 protein: N-terminal RNA binding domain (RBD, 1–73 aa) and C-terminal effector domain (ED, 74–230/237 aa) [5–7]. These two domains function in viral infection through protein-RNA and protein–protein interactions with double-stranded RNA and certain viral proteins [8, 9]. NS1 protein is a multifunctional regulatory protein and plays an important role in regulating the pathogenicity and virulence of the avian influenza virus [10, 11]. NS1 interacts with a variety of host proteins in the cells, including Staufen protein, eukaryotic translation initiation factor 4GI (eIF4GI), NS1 binding factor (NS1-I), poly (A) binding protein I (PABPI), and P85β protein [12–16]. Through protein–protein interaction, NSl protein can reinforce the transcription and translation of viral mRNA, inhibit the synthesis of host protein, change the distribution of host proteins, and restrain production of interferon. It can also reduce the host’s apoptosis and anti-virus capabilities, and promote the replication and release of virus particles. Therefore, research on the interaction between NSl and host proteins is expected to further reveal the role of NSl in AIV pathogenesis.

In order to find more host proteins that interact with NS1, T7-phage display system was used to screen a lung cell cDNA library for potential NS1-interacting protein. One of the positive clones obtained from this experiment was identified as nucleolar and coiled-body phosphoprotein 1(NOLC1), which is a highly phosphorylated protein that exists in the interphase of the nucleolus [17] and can bind to RNA polymerase I to regulate the transcription of rRNA during mitotic division. NOLC1 plays an important role in the regulation of cell growth, inflammation genesis, and hepatoma development [18]. Co*-*immunoprecipitation and His-pull experiments were used to further investigate the interaction between NS1 and NOLC1. In addition, fusion proteins consisting of NS1-green fluorescent protein and of NOLC1-red fluorescent protein co-expressed in HeLa cells were found to be co-localized in the nucleus. Furthermore, gene truncating experiments revealed that the effector domain of NS1 was sufficient to interact with NOLC1. This study lays the foundation for furthering our understanding of the interaction between NS1 and host cells, and the replication mechanism of avian influenza virus in the host cells. Given the important roles of NOLC1, this finding also provides a potential target for anti-influenza drug design. This would be particularly relevant for prophylaxis and treatment in the event of an emerging influenza virus outbreak.

## Materials and methods

### Materials

#### Cell lines, vectors, bacterial strains

A/Goose/Guangdong/1/96 H5N1 influenza virus and human embryonic kidney cell line 293T cells were obtained from a culture stored in the State Key Laboratory of virology of Wuhan University. pET-28a plasmid, pCMV-flag plasmid, *E. coli* DH5α, and *E.coli* BL21 competent cells were maintained in our laboratory.

#### Reagents

DNA Marker DL2000, restriction enzymes *Eco*RI and *Bam*HI were purchased from Takara. Protein marker was purchased from MBI. High purity plasmid (small) extraction kit was purchased from Beijing Union International Biological Gene Inc; isopropyl-β-D-thiogalactopyranoside (IPTG) was purchased from Beijing Bio-Tech Gene Technology LLC. Ni–NTA His-bind Resins were obtained from Novagen Inc. Lipofectamine™ 2000 was purchased from Invitrogen Corporation. PMSF (protease inhibitor) was purchased from Amersco Corporation. Protease cocktail was obtained from Merck Corporation; Protein A/G-plus-Agarose beads from Promega Company; OctA-Probe antibody from Santa Cruz Corporation; MagneHis™ Protein Purification System from promega; Horseradish peroxidase labeled secondary antibody from Beijing Zhongshan Golden Bridge Company; and Western blotting Luminol Reagent test kit from Santa Cruz Corporation.

### Methods

#### Screening of NS1-interacting host protein using T7-phage display system

The pET-28a-NS1 construct was made by inserting the corresponding cDNA fragment derived from the A/Goose/Guangdong/1/96 H5N1 influenza virus into the *Bam*HI/*Eco*RI sites of pET-28a. The recombinant NS1 was expressed in *E. coli* BL21(DE3). After expression the cells were harvested, 5 mL solution A (10 mmol/L Tris–HCl, pH 8.0), 20–30 mL binding buffer containing 10 mmol/L imidazole, 1.5 mL of 100 μg/mL lysozyme, and 30 μL of 1 mmol/L PMSF were added to the cells and mixed ad subjected to two freeze/thaw cycles at −70 °C/room temperature. The cells were disrupted by an ultrasonic cell disruptor (200–300 W output power) while being kept in an ice bath. After centrifugation (10,000×*g*, 15 min), the NS1 protein in the supernatant was purified by Novagen Ni–NTA His-bind Resins as described previously [19]. Microtiter plate wells were coated with 1 μg NS1 protein dispensing 100 μL PBS containing 0.01 μg/mL NS1 onto each well and the plate was incubated at 4 °C for overnight. Human liver cell cDNA Phage library was added and incubated for 2 h at RT. After washing with PBS containing 0.1 % Tween 20 for 10 times, the adsorbed phage particles were eluted with 1 % SDS solution and then amplified by infection of *E.coli* BLT5403 to generate sub-libraries. This binding-elution-amplification procedure was repeated 3–5 rounds until the eluted phage titer was stabilized at a certain titer. After round 5, 24 positive plaques were randomly picked, the phage genome was extracted, and the inserted segment of the phage DNA was amplified by PCR using T7 select up and down primers. The amplified products were detected by 1 % agarose gel electrophoresis, purified and subjected to DNA sequencing. The DNA sequences obtained were analyzed by blast in NCBI.

#### Affinity-binding assays by co-immunoprecipitation (Co-IP)

The recombinant plasmids pEGFP-N1-NS1 and pCMV-flag-NOLC1 were constructed and co-transfected into 293T cells. After 48 h, the transfected cells were collected and washed three times in PBS, harvested by scraping, and centrifuged for 5 min at 500×*g*. The cell pellet was homogenized at 4 °C by pipetting up and down with 1 mL of cell lysis buffer. The homogenate was centrifuged at 14,000×*g* for 20 min at 4 °C, and the supernatant was incubated with 5 μL of anti-FLAG polyclonal antibody and 20 μL protein A/G-plus beads for overnight at 4 °C. The resins were recovered by centrifugation at 700×*g* for 5 min and washed three times with cell lysis buffer followed by centrifugation after each wash. The pellet was then washed twice with 50 mM Tris–HCL (pH 7.5) containing 0.1 % (w/v) SDS and 150 mM NaCl. After the final wash, it was resuspended in 60 μL 1× SDS loading buffer, heated at 100 °C for 3 min and subjected to SDS-PAGE followed by western blot analysis with anti-GFP antibody. As a control, EGFP-NS1 and pCMV-flag were also co-transfected into 293T cells and subjected to the same experiment.

#### Affinity-binding assays by pull-down experiment

His-NS1 fusion protein was expressed in *E. coli* BL21 induced by IPTG. Five milligrams of His-NS1 was adsorbed onto MagneHis™ beads, as recommended by the manufacturer (Promega). The immobilized proteins were mixed with 200 μL of 293T cell extracts containing flag-tagged NOLC1 in binding buffer (50 mM Tris–HCl, 150 mM NaCl, 1.0 %Triton X-100, pH 7.6) containing a protease inhibitor mix (Roche) and incubated for 1 h at room temperature on a rotator. Beads were precipitated and washed three times with 500 μL of binding buffer. Proteins bound to His-tagged-NS1 were analyzed by SDS-PAGE, followed by western blot analysis with anti-flag antibody.

#### Subcellular localization by confocal microscopy

The recombinant plasmids pEGFP-N1-NS1 and pDsRed-C1-NOLC1 were constructed and co-transfected into Hela cells at a ratio of 1:3 for pEGFP-N1-NS1: pDsRed-C1-NOLC1 using Lipofectamine™ 2000. For controls, pDsRed-C1 and pEGFP-N1 were co-transfected into Hela cells instead. The transfected cells were grown on glass coverslips. Two days after transfection, the cells were fixed with 4 % paraformaldehyde in phosphate-buffered saline (PBS) for 20 min at room temperature and the nuclei were stained with DAPI. The distribution and localization of the green fluorescence, red fluorescence, and the cell nuclei were observed by confocal microscope.

#### Identification of interaction domains by gene truncation and co-immunoprecipitation

In order to locate the region of NS1 that interact with the protein identified from the host cells, eukaryotic expression vectors pEGFP-N1-NS1-RBD(1–73 aa) and pEGFP-N1-NS1-ED(74–230 aa), which express the N-terminal RNA binding domain and C-terminal effector domain of NS1 protein, respectively, were constructed. The protein–protein interactions between truncated NS1 and NOLC1 were investigated by co-immunoprecipitation as described above.

## Results

### Phage display screening

**Fig. 1:**
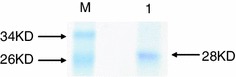
SDS-PAGE analysis of purified NS1. Expression of NS1 by *E. coli* BL21(DE3) harboring pET-28a-NS1 was induced by 1 mmol/L IPTG. The cells were harvested, and soluble fraction of the lysate was subjected to protein purification via Novagen Ni–NTA His-bind Resins and the purified protein was analyzed by SDS-PAGE. M, Protein Marker; *lane 1*, Purified NS1

*Escherichia coli* BL21 strain harboring pET-28a-NS1 was cultured and induced by IPTG to express NS1 protein. Cell extract was prepared and soluble proteins of the extract were collected and subjected to SDS-PAGE analysis. A specific band corresponding to 28 kDa was detected in the gel (Fig. 1). In order to screen the proteins that interact with NS1, five rounds of bio-screening were performed. The titers of phages that bound to NS1 increased after each round of screening, which suggested that clones of higher affinity were enriched. Overall, 104-fold increase in recovery rate was obtained after five rounds of screening. Twenty-four plaques were randomly selected from the fifth round of screening and the inserted segment was sequenced and blasted against NCBI database. The results showed that the protein that interacted with NS1 was the Human NOLC1.

### Interaction between NS1 and NOLC1 as determined by immunoprecipitation

**Fig. 2:**
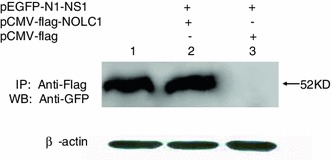
Interaction between NS1 and NOLC1 as determined by immunoprecipitation. pEGFP-N1-NS1 and pCMV-flag-NOLC1 were co-transfected into the 293T cells, and the cell lysate were subjected to immunoprecipitation with anti-FLAG polyclonal antibody. The immunoprecipitated proteins were detected by western blot with anti-GFP antibody. *Lane 1*, NS1 as detected by western blot with anti-GFP antibody; *lane 2* and *lane 3*, IP performed with the appropriate transfection as indicated followed by western blot. Beta-actin was detected from whole cell lysate

We further confirmed the interaction between NOLC1 and NS1 using immunoprecipitation. The expression vectors pEGFP-N1-NS1 and pCMV-flag-NOLC1 were co-transfected into 293T cells. Twenty-four hours after transfection the cell extracts were prepared and subjected to immunoprecipitation with anti-flag antibody. The precipitated proteins were analyzed by western blot with anti-GFP antibody. The results showed that NOLC1 was associated with NS1 protein. However, no band was detected when cells expressed NS1 without NOCL1 (Fig. 2).

### NS1 and NOLC1 interaction determined by pull-down assay

**Fig. 3:**
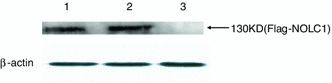
NS1 and NOLC1 interaction determined by pull-down assay. *Lane 1*, Lysate from 293T cells expressing flag-NOLC1was subjected to direct western blot with anti-flag anitbody. Lysate from 293T cells expressing flag-NOLC1was incubated with His-tagged NS1-MagneHis™ beads (*lane 2*) or just MagneHis™ beads (*lane 3*) followed by western blot of the bead-bound proteins with anti-flag antibody

Pull-down was also performed to further confirm the interaction between NOLC1 and NS1. The His-NS1 fusion protein expressed in *E. coli* BL21 were adsorbed to MagneHis™ beads and then incubated with the lysate of 293T cell overexpressing Flag-NOLC1 fusion protein. Proteins bound to His-NS1 were analyzed by western blot with anti-flag antibody, and a band at approximately 130 kD was detected as expected. This band was not detected in the negative control, in which lysate of 293T cells overexpressing Flag-NOLC1 fusion protein was incubated with just MagneHis™ beads (Fig. 3).

### Co-localization of NOLC1 and NS1 proteins

**Fig. 4:**
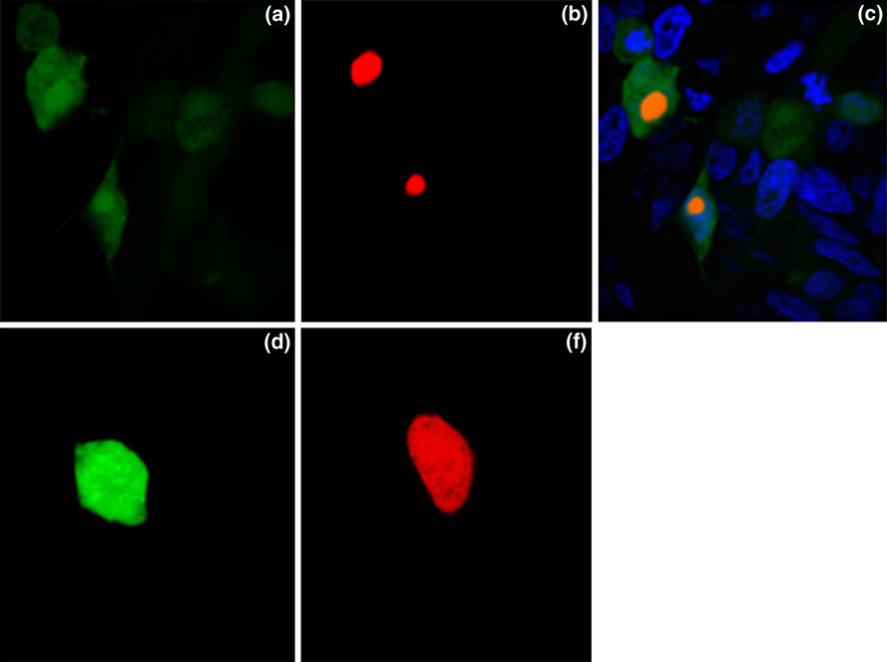
Subcellular localization of NOLC1 and NS1. HeLa cells were co-transfected with NOLC1-DsRed and NS1-EGFP (**a**, **b**, **c**) or with DsRed and EGFP only (**d**, **e**). Cells were detected by *green fluorescence* (**a**, **d**), *red fluorescence * (**b**, **e**), or DAPI staining, and the image of which was merged with those of **a** and **b** (**c**) (Color figure online)

Based on the results above, we hypothesized that the interaction between NOLC1 and NS1 is physiologically relevant, so we expected the two proteins to occupy the same intracellular compartment. To confirm this prediction, DsRed-NOLC1 and EGFP-NS1 fusion proteins were co-expressed in HeLa cells. EGFP-NS1 fusion protein was mainly localized in the nucleus but a small amount of it was also distributed in the cytoplasm, whereas DsRed-NOLC1 protein was localized only in the nucleolus. The results showed that NS1 and NOLC1 were co-localized in the nuclear compartment (Fig. 4).

### Identification of interaction domains

**Fig. 5:**
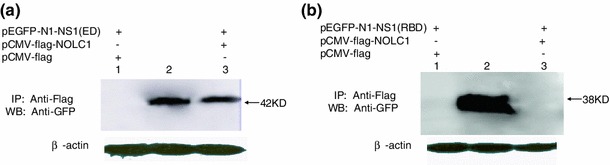
Interaction between domain of NS1 and NOLC1. pEGFP-N1-NS1-RBD and pEGFP-N1-NS1-ED, which express the N-terminal RNA binding domain and C-terminal effector domain of NS1, respectively, were coexpressed with NOLC1 in 293T cells. The protein–protein interactions between truncated NS1 proteins and NOLC1 were investigated by coimmunoprecipitation followed by western blot. **a** RBD domain plus NOLC1. *Lane 1*, NOLC1and RBD as determined by IP and western blot; *lane 2*, Input of RBD (western blot only); *lane 3*, GFP and RBD as determined by IP and western blot. **b** ED domain plus NOLC1. *Lane 1*, NOLC1 and ED as determined by IP and western blot; *lane 2*, Input of ED (western blot only); *lane 3*, GFP and ED as determined by IP and western blot. Beta-actin was detected using a whole cell lysate

Nonstructural protein 1 consists of two major domains, the N-terminal RNA binding domain (RBD) and the remaining effector domain (ED). In order to determine which domain of NS1 interacts with NOLC1, each of the two domains of NS1 were separately constructed and expressed followed by Co-IP analysis. The results showed that the effector domain of the NS1 was the domain that interacted with NOLC1 (Fig. 5).

## Discussion

NS1 is one of the known non-structural proteins encoded by the avian influenza virus that plays an important role in the regulation of the pathogenicity and virulence of influenza virus; it might be the key to the high mortality rate associated with avian influenza. As a multifunctional protein, NS1 can interact with a variety of host cell proteins to weaken the host’s antiviral resistance and strengthen the virus’ propagation. Previous researches have shown that NS1 can inhibit the expression of host cell proteins, thereby strengthening the synthesis of viral proteins and inhibiting the production of interferon. In addition, NS1 protein can regulate the apoptosis of host cells to ensure the virus’ efficient replication in the cells [20]. In depth study of NS1 not only allows us to better understand the interaction between the host cell and the virus, but also provides us with a theoretical basis of the relationship between the NS1 and the virulence of the virus.

In this study, we found a new protein that interact with NS1 through T7-phage display technology, and identified it as NOLC1, which was previously named as Nopp140 or p130. Co-IP and His-pull-down experiments confirmed the specific interaction between these two proteins. NOLC1 protein is a highly phosphorylated protein that exists in the interphase nucleolus [17]. It can itself distribute uniformly in cytoplasm, and function both as a chaperone that shuttles between the nucleolus and cytoplasm, as well as a regulator which binds to RNA polymerase I to regulate the transcription of rRNA during mitotic division. NOLC1 is thought to be involved in the activation carried out within the nucleolus. It is also a mediator of protein kinase A signaling pathway that activates the acute phase response a1_acid glycoprotein gene [21]. Recent results have shown that NOLC1 may play an important role in the regulation of cell growth, inflammation genesis, and hepatoma development [18]. It plays a role in the regulation of tumorigenesis of nasopharyngeal carcinoma progression (NPC), both NOLC1 and tumor protein 53 work in synergism to activate the MDM2 promoter in NPC cells [22]. These observations suggested that NOLC1 was crucial for normal cell growth. So we proposed that when the influenza virus infects the host cells and express its protein, the NS1 will bind to and inactivate NOLC1, losing a crucial protein for its normal cell growth, and at the same time undergo apoptosis. That is one way through which the cell can resist viral infection.

Our study also showed that NS1 was mainly localized in the nucleus, while DsRed-NOLC1 was localized only in the nucleolus. The two proteins occupied the same intracellular compartment, suggesting that the interaction between NOLC1 and NS1 was physiologically relevant. To further investigate the interaction of these two proteins, the domain of NS1 that was involved in the interaction with NOLC1 was mapped through gene truncation experiment, which identified the effector domain (ED) as the domain that interacted with the NOLC1. The ED interacts with cellular targets such as Staufen protein, eukaryotic translation initiation factor 4GI (eIF4GI), NS1-I, PABPI, and P58β protein, and contributes to the virulence of AIV. Our results have shed more light into the function of the effector domain of NS1. Future work will attempt to indentify the specific region within the effector domain that interacts with NOLC1 through additional gene truncation as well as point mutation aiming to provide a new target spot for the screening of anti-viral drugs. Further investigation will also be carried out to examine the effect of the interaction between NOLC1 and NS1 on the functional activity of host cells and the cell cycle, which will provide the theoretical basis for better understanding of the function of NS1 and the mechanism associated with the pathogenesis of AIV.
